# Influence of Bioactive, Inorganic, and Organic Fillers on Basic Properties of 4-META/MMA-TBB Resin as Restorative Materials for Root Caries

**DOI:** 10.3290/j.jad.c_2428

**Published:** 2025-12-16

**Authors:** Miho Kikuta, Masanao Inokoshi, Rena Takahashi, Mao Yamamoto, Hiraku Onuma, Kumiko Yoshihara, Manabu Kanazawa

**Affiliations:** a Miho Kikuta Doctoral Student, Department of Gerodontology and Oral Rehabilitation, Graduate School of Medical and Dental Sciences, Institute of Science Tokyo, 1-5-45 Yushima, Bunkyo, Tokyo 113-8549, Japan. Conceptualized and designed the study, conducted the experiments, analyzed the data, and drafted the manuscript.; b Masanao Inokoshi Full Professor, Department of Oral Devices and Materials, Graduate School of Medical and Dental Sciences, Institute of Science Tokyo; Section Head, Oral Science Center, Institute of Biomedical Engineering, Institute of Science Tokyo, 1-5-45 Yushima, Bunkyo, Tokyo 113-8549, Japan. Supervised the project, provided scientific guidance throughout the study, and critically revised the manuscript.; c Rena Takahashi Junior Associate Professor (Career Track), Department of Cariology and Operative Dentistry, Graduate School of Medical and Dental Sciences, Institute of Science Tokyo, 1-5-45 Yushima, Bunkyo, Tokyo 113-8549, Japan. Provided technical guidance on the experimental process and approved the final version of the manuscript.; d Mao Yamamoto Adjunct Lecturer, Department of Gerodontology and Oral Rehabilitation, Graduate School of Medical and Dental Sciences, Institute of Science Tokyo, 1-5-45 Yushima, Bunkyo, Tokyo 113-8549, Japan. Provided technical guidance on the experimental process and approved the final version of the manuscript.; e Hiraku Onuma Assistant Professor, Department of Gerodontology and Oral Rehabilitation, Graduate School of Medical and Dental Sciences, Institute of Science Tokyo, 1-5-45 Yushima, Bunkyo, Tokyo 113-8549, Japan. Provided technical guidance on the experimental process and approved the final version of the manuscript.; f Kumiko Yoshihara Senior Researcher, National Institute of Advanced Industrial Science and Technology (AIST), Health Research Institute, 2217-14 Hayashi-cho, Takamatsu, Kagawa 761-0395, Japan. Provided technical guidance on the experimental process and approved the final version of the manuscript.; g Manabu Kanazawa Full Professor, Department of Gerodontology and Oral Rehabilitation, Graduate School of Medical and Dental Sciences, Institute of Science Tokyo, 1-5-45 Yushima, Bunkyo, Tokyo 113-8549, Japan; Visiting Guest Professor, University of Zürich, Rämistrasse 71, CH-8006 Zürich, Switzerland. Supervised the overall project, provided guidance during the study, and approved the final version of the manuscript.

**Keywords:** 4-META/MMA-TBB resin, bioactive glass, mineralization ability, root caries, shear bond strength, three-point bending strength

## Abstract

**Purpose:**

This study aimed to evaluate the effects of bioactive, inorganic, and organic fillers on the curing time, mechanical properties, mineralization ability, and shear bond strength to dentin of 4-methacryloxyethyl trimellitate anhydride/methyl methacrylate-tri-n-butylborane (4-META/MMA-TBB) resin-based restorative materials.

**Materials and Methods:**

Experimental resins were prepared by incorporating 3 wt% organic composite filler, bioactive glass, or aluminosilicate glass into 4-META/MMA-TBB resin. A commercial organic-filler-containing 4-META/MMA-TBB resin (Bondfill SB Plus (BSP)) was also tested for comparison. The curing time, three-point flexural strength, and shear bond strength to root dentin were measured and statistically analyzed with a significance level of α = 0.05. The mineralization ability of groups containing bioactive glass was evaluated based on the formation of hydroxyapatite in simulated body fluid.

**Results:**

The bioactive glass group exhibited prolonged curing times and reduced flexural strengths compared to BSP. Bioactive glass induced slight mineralization. Organic composite filler reduced the curing time and significantly increased the shear bond strength to dentin. After thermal cycling, the organic composite filler groups retained higher bond strengths than the other groups.

**Conclusion:**

Incorporating organic composite fillers into 4-META/MMA-TBB resin may improve clinical handling by reducing the curing time while maintaining or enhancing the bond strength to dentin, suggesting their suitability for restorative applications, particularly in moisture-compromised environments.

In recent years, population aging and improved dental care have led to an increased number of retained teeth among older adults.^1^ However, in a 1-year follow-up study of 186 dental patients aged 65 years or older in Okayama Prefecture, Japan, a majority (59.6%) had at least one root surface carious lesion.^24^ Similarly, a survey conducted in Hubei Province, China, involving 1080 participants aged 35–44 or 65–74 years from both urban and rural areas, reported a 13.1% prevalence of root caries in the middle-aged group and 43.9% among older adults.^8^ These findings indicate that the prevalence of root caries increases with age and has become a growing concern in clinical dentistry.

Treating root caries in older adults presents several clinical challenges. This population is susceptible to gingival recession, plaque accumulation on root surfaces, and gingival bleeding associated with periodontitis, which complicates moisture control in the operative field.^12^ Additionally, older adults with cognitive impairment may struggle to understand and follow dental instructions, exacerbating the difficulty of maintaining a dry working environment.^15^ Moisture negatively affects the bonding efficacy and mechanical properties of restorative materials, which may lead to marginal degradation, microleakage, and eventual debonding of the restoration. These issues can ultimately result in recurrent caries or restoration failure.^3,16^

In this context, restorative materials must be carefully selected for managing root caries in older adults, necessitating a thorough understanding of their properties. Composite resins and glass ionomer (GI) cements are widely used in clinical practice. Composite resins are preferred owing to their favorable mechanical properties and esthetic qualities. However, achieving adequate adhesion with composite resins requires a dry environment.^12,24^ As a result, GI cements are commonly used for restorations in older adults. GI cements are favored for their biocompatibility, chemical bonding to dental hard tissues, and fluoride release, all of which may contribute to the prevention of recurrent caries.^4^ Nevertheless, GI cements generally exhibit lower bond strengths to dentin than resin-based materials and are highly sensitive to moisture during the setting process, which may compromise their clinical performance. Furthermore, conventional GI cements have limited mechanical properties, including relatively low flexural and tensile strengths.^2^

Recently, resin-based restorative materials such as 4-methacryloxyethyl trimellitate anhydride/methyl methacrylate-tri-n-butylborane (4-META/MMA-TBB) have gained attention as promising alternatives to conventional composite resins and GI cements. These materials have superior bonding performance to conventional composite resins^21^ and are particularly effective in moisture-compromised environments.^19^ Furthermore, research has shown that 4-META/MMA-TBB resins have long-term bonding durability.^28^ However, the effects of reinforcing fillers and bioactive agents on their physical and functional properties remain poorly understood.

This study investigates the effects of organic composite filler, bioactive glass, and aluminosilicate glass on the physical properties of 4-META/MMA-TBB resin-based restorative materials. These fillers were selected because previous reports have indicated that they may improve the physical properties of resin-based restorative materials: organic composite fillers may provide mechanical reinforcement; bioactive glasses exhibit ion-releasing and remineralizing potential; and aluminosilicate glasses may improve the flexural strength. The curing time, mechanical properties, and shear bond strength to root surface dentin were evaluated and compared with those of conventional materials. In addition, the mineralization ability of materials with bioactive glass was explored. The null hypothesis was that organic composite filler, bioactive glass, and aluminosilicate glass have no effect on the curing time, mechanical properties, or shear bond strength of 4-META/MMA-TBB resin-based restorative materials. The second null hypothesis was that bioactive glass has no effect on the mineralization ability of 4-META/MMA-TBB resin-based restorative materials.

## MATERIALS AND METHODS

**Fig 1:**
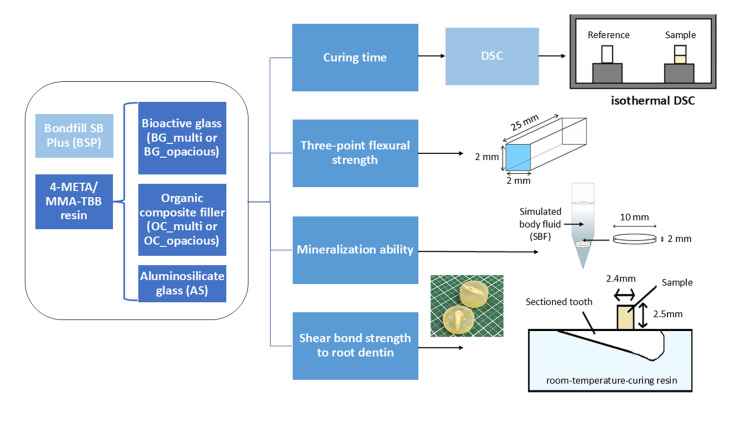
Experimental design: prepared specimens (left), evaluation methods (center), and test specimens (right).

An overview of the experimental design is shown in Figure 1.

### Materials

Experimental groups were prepared by adding 3 wt% of filler to 4-META/MMA-TBB resin. The fillers used were: organic composite filler (OC, multi or opacious; radiopaque polymer-coated filler, particle size: 15 μm, density: 7.0 g/cm^3^), bioactive glass (BG, multi or opacious; 45S5, particle size: 5 μm, density: 2.8 g/cm^3^), and aluminosilicate glass (AS; particle size: 1 μm, density: 2.4 g/cm^3^). The volume fractions (vol%), as calculated based on the nominal weight fractions (3 wt%), powder compositions, and filler density, were 6.84 vol% for OC, 1.61 vol% for BG, and 1.87 vol% for AS. The multi and opacious variants of the organic composite filler and bioactive glass differed only in pigment composition, which accounted for less than 1 wt% of the total formulation; the remaining components (>99 wt%) were identical. A commercial organic-filler-containing 4-META/MMA-TBB resin (denoted as BSP; Bondfill SB Plus, Sun Medical, Shiga, Japan) was also tested for comparison. The number of samples was determined with reference to previous literature (curing time: n = 3 per group^5,23^; three-point flexural strength and shear bond strength: n = 15 per group^25^; mineralization ability: n = 10 per group^20^).

### Curing Time

The curing behavior of the 4-META/MMA-TBB resin-based materials was evaluated using isothermal differential scanning calorimetry (DSC; DSC-60, Shimadzu Corporation, Kyoto, Japan). The resin was cured by heating within the DSC instrument at a constant temperature of 37°C. Because 4-META/MMA-TBB resin is not photocurable, no light-curing unit was used. The time at which the maximum exothermic peak appeared during the polymerization process was taken as the curing time.

### Three-point Flexural Strength

The flexural strength was assessed using three-point bending tests. Building on previous studies,^25^ the 4-META/MMA-TBB resin-based materials were incrementally applied to Teflon molds using a brush to create rod-shaped specimens (2 mm × 2 mm × 25 mm) in accordance with ISO 4049. The specimens were stored at 37°C and 100% relative humidity for 1 week, after which they were tested using a universal testing machine (EZ-LX, Shimadzu Corporation, Kyoto, Japan) at a crosshead speed of 1 mm/min. The span length was set at 20 mm.

### Evaluation of Mineralization Ability

Mineralization tests were performed for the BSP, BG_multi, and BG_opacious groups. Disk-shaped specimens (10 mm diameter × 2 mm thickness) were prepared by incrementally applying the resin to a mold using a brush. The specimens were then immersed in simulated body fluid (SBF), prepared according to ISO 23317, and incubated at 37°C for 4 or 8 weeks. The deposition of crystalline phases on the surfaces of the specimens was assessed. The surface morphology and elemental composition were analyzed by scanning electron microscopy (SEM; JSM-7900F, JEOL, Tokyo, Japan) and energy-dispersive X-ray spectroscopy (EDS; JED-2300, JEOL). Before the EDS analysis, the surfaces of the specimens were coated with carbon. SEM observations were conducted at an accelerating voltage of 15.0 kV and a current of 7.475 nA. Semi-quantitative elemental analysis was performed on the crystalline deposits observed on the specimen surfaces. In addition, grazing incidence X-ray diffraction (GIXRD; PANalytical X’pert PRO MPD, PANalytical, Almelo, Netherlands) was performed with Cu Kα radiation (45 kV, 40 mA). The measurements were conducted with a fixed incidence angle of 1.0°, 2θ scanning range of 2°–55°, step size of 0.02°, and time per step of 1.00 s.

### Shear Bond Strength to Root Surface Dentin

The shear bond strength to root surface dentin was evaluated using extracted human teeth. To ensure consistency across specimens, only teeth with sound, intact root dentin were used. In addition, all carious tissue was removed, because controlling the quality of specimens is difficult with selective caries removal. This approach is consistent with previous studies^25^ and allows reliable shear bond strength evaluation. Ethical approval for the use of human teeth was granted by the Ethics Review Committee of the Faculty of Dentistry at the Institute of Science Tokyo (D2016-025). After the periodontal tissue was removed, the teeth were stored in a 0.5% chloramine T solution (chloramine T trihydrate, Tokyo Kasei Kogyo Co., Tokyo, Japan). The teeth were sectioned sagittally using a precision cutting machine Preciso CL40, Sankei, Tokyo, Japan) and embedded in a room-temperature-curing resin (Unifast III Clear, GC, Tokyo, Japan). The exposed root dentin surfaces were flattened using a trimmer (Y-230, Yoshida, Tokyo, Japan) and then polished with waterproof abrasive paper (DCCS-600, Sankyo Rikagaku Co., Saitama, Japan).

Following the manufacturer’s instructions, a primer (Teeth Primer, Sun Medical) was coated on the root dentin for 20 s and air-dried using a three-way syringe. A mixture of base and catalyst was then applied to the bonding surface to enhance the wettability. A cylindrical mold (Bonding Mold Insert, Ultradent Products, South Jordan, UT, USA) with an internal diameter of 2.4 mm and a height of 2.5 mm was used to apply the resin to the dentin surface. The specimens were allowed to cure at room temperature for 30 min before demolding.

The specimens were divided into two groups: one group was stored in ultrapure water at 37°C for 24 h prior to testing (24 h group), while the other underwent thermal cycling (TC group) by alternately immersing the specimens in water baths at 5 ± 1 and 55 ± 1°C (K178-08, Tokyo Giken, Tokyo, Japan), with an immersion time of 30 s and transfer time of 2 s between baths. This process was repeated for a total of 10,000 cycles. The samples were then subjected to shear bond strength tests using a universal testing machine (EZ-LX, Shimadzu Corporation, Kyoto, Japan) at a crosshead speed of 1 mm/min.

Following the shear bond tests, the fracture surfaces were examined by optical microscopy (Stemi 305, ZEISS, Jena, Germany). The fracture modes were classified as adhesive failure, cohesive failure, or mixed failure.

### Statistical Analysis

Depending on the results of Shapiro–Wilk’s test for normality, the curing time was analyzed using either one-way analysis of variance (ANOVA) followed by Tukey’s post hoc test, or Kruskal–Wallis’s test followed by Dunn’s post hoc test. The flexural strength data were evaluated by Weibull analysis using the maximum likelihood estimation (MLE) method implemented in the WeibullR package, with confidence intervals calculated by the likelihood ratio method; statistical differences between Weibull distributions were assessed by comparing likelihood contour lines. The shear bond strength was analyzed using a linear mixed-effects model, treating individual teeth as random effects. All analyses were performed in R (version 4.3.1, R Foundation for Statistical Computing, Vienna, Austria), with the level of significance set at α = 0.05.

## RESULTS

### Curing Time

**Fig 2:**
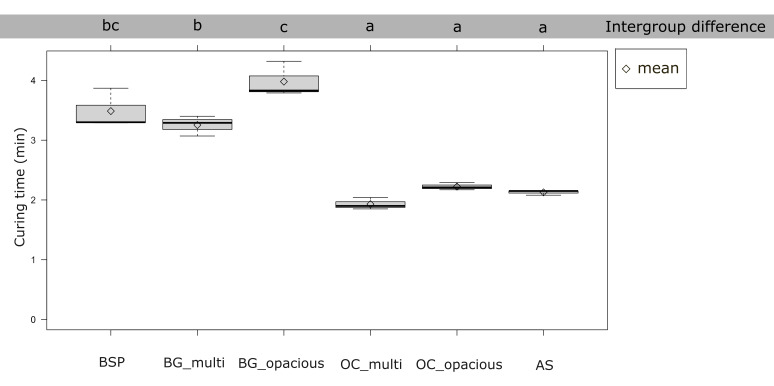
Curing time of 4-META/MMA-TBB resin, as measured by DSC. Different lowercase letters indicate significant differences (α < 0.05).

The curing time results are presented in Figure 2. The OC_multi (P < 0.001), OC_opacious (P < 0.001), and AS (P < 0.001) groups exhibited significantly shorter curing times than the BSP, BG_multi, and BG_opacious groups. BG_opacious showed a significantly longer curing time than BG_multi (P = 0.00808). No significant differences in curing time were observed among the OC_multi, OC_opacious, and AS groups (P > 0.05), or among the BG_multi, BG_opacious, and BSP groups (P > 0.05).

### Three-point Flexural Strength

**Fig 3a and b:**
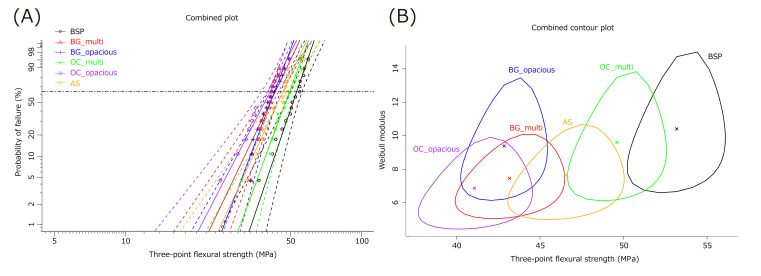
Weibull analysis of three-point flexural strength. (a) Weibull plot; (b) Weibull contour plot. The circles in the contour plot represent 95% confidence intervals for each group; a lack of overlap indicates statistically significant differences.

The three-point flexural strength results are presented in Figure 3. The OC_opacious, BG_multi, BG_opacious, and AS groups exhibited significantly lower flexural strengths than BSP. OC_multi also showed lower flexural strength than BSP; however, the difference was not significant. Although no significant differences were observed between the AS group and the BG_multi, BG_opacious, or OC_opacious groups, the AS group exhibited slightly higher flexural strength.

### Mineralization Ability

**Fig 4a and b:**
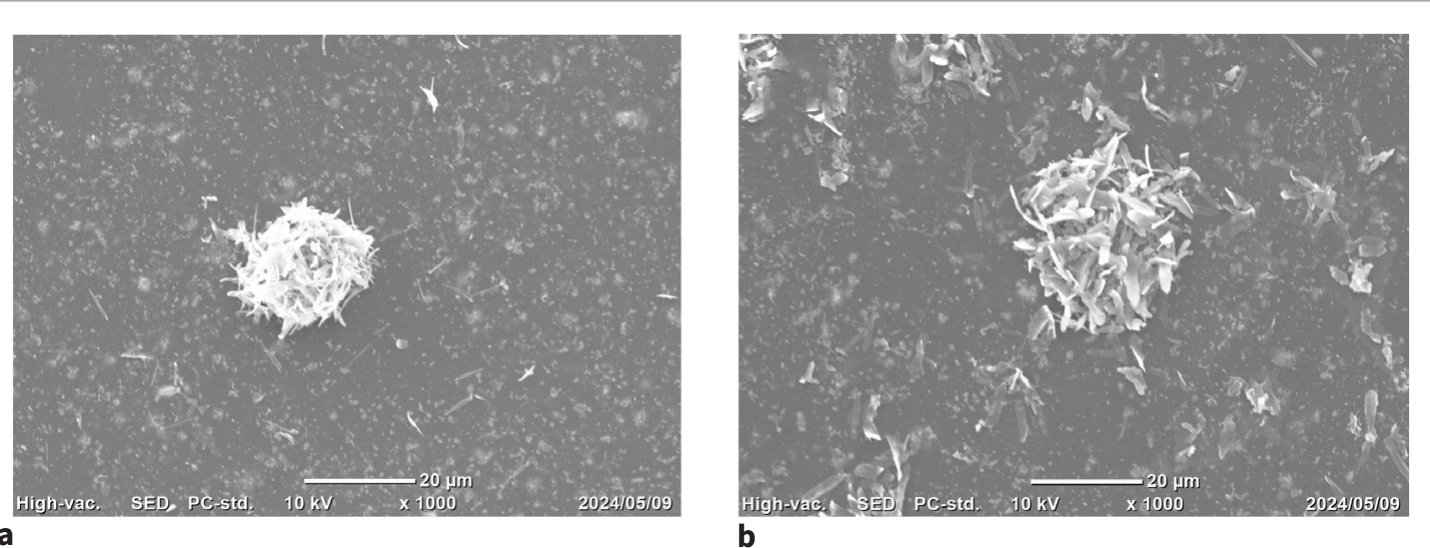
SEM images of (a) BG_multi and (b) BG_opacious groups showing needle-like crystals on the specimen surface.

**Fig 5a to f:**
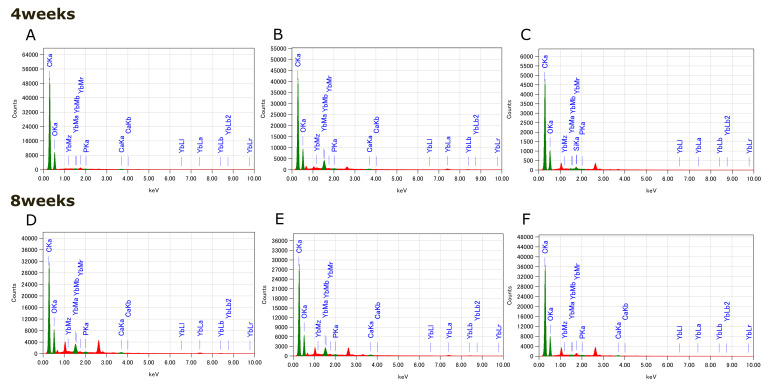
EDS spectra of (a, d) BSP, (b, e) BG_multi, and (c, f) BG_opacious groups after (a to c) four and (d to f) 8 weeks. Only trace amounts of Ca and P were detected for all groups.

The mineralization results for the BSP, BG_multi, and BG_opacious groups are presented in Figures 4 and 5. EDS analysis conducted at 4 and 8 weeks revealed prominent peaks corresponding to C and O, as well as multiple M and L lines attributed to Yb. In contrast, peaks corresponding to Ca and P, which are key components of hydroxyapatite, were extremely weak. No notable differences in Ca or P content were observed among groups. SEM imaging revealed needle-like crystalline structures on the surfaces of some specimens in the BG_multi and BG_opacious groups. However, GIXRD analysis did not detect crystalline hydroxyapatite peaks.

### Shear Bond Strength To Root Surface Dentin

**Fig 6:**
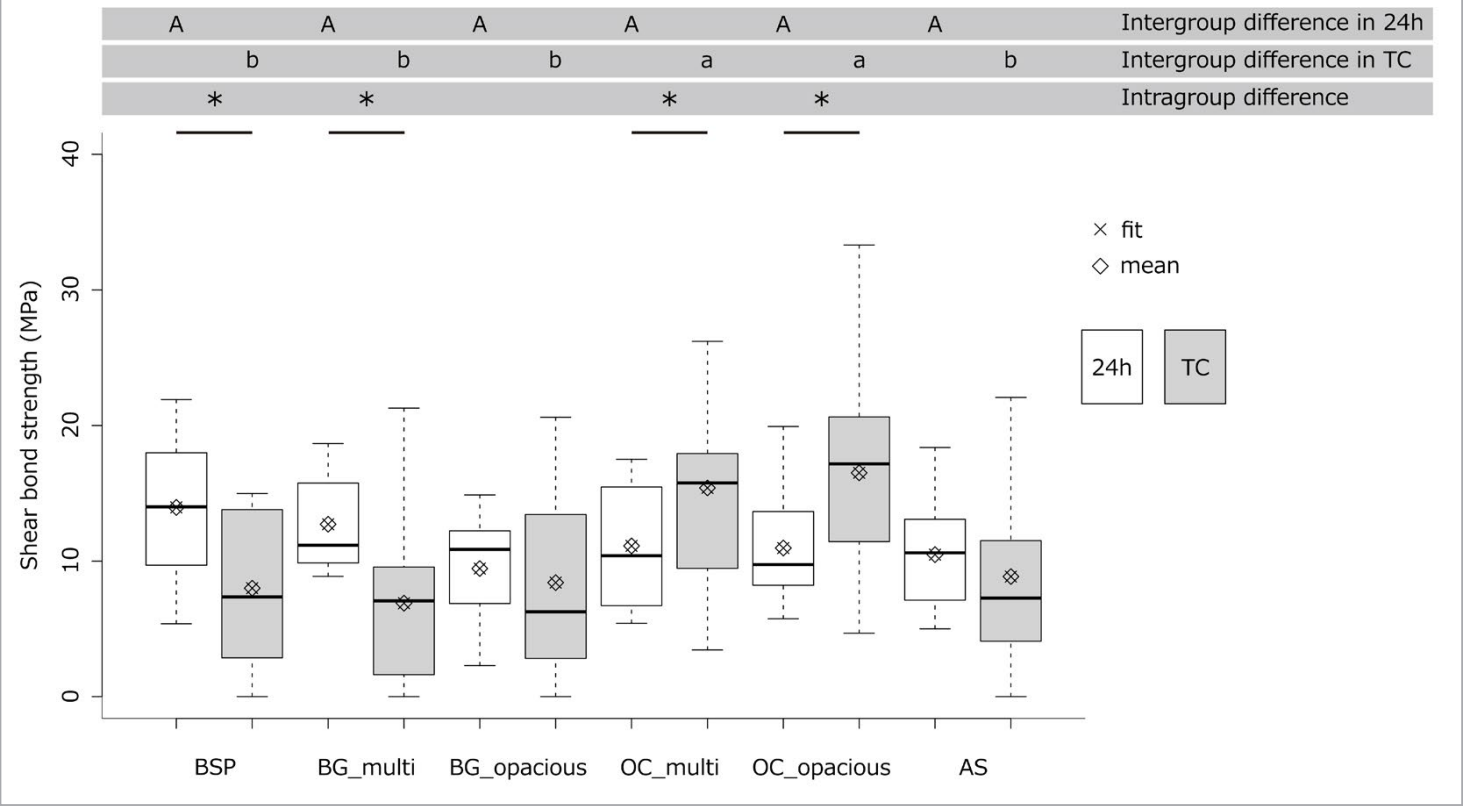
Analysis of shear bond strength to root dentin using linear mixed-effects model. Different letters or asterisks indicate significant differences (α < 0.05). “Fit” refers to the predicted values generated by the linear mixed-effects model.

The results of the shear bond strength tests are presented in Figure 6. After immersion in ultrapure water for 24 h at 37°C, no significant differences in shear bond strength were observed among groups (P > 0.05). In contrast, after thermal cycling for 10,000 cycles between 5 and 55°C, the OC_multi (P < 0.05) and OC_opacious (P < 0.05) groups exhibited significantly higher shear bond strengths than the other groups. Intragroup comparisons between the 24 h immersion and thermal cycling conditions revealed a significant decrease in shear bond strength after thermal cycling for the BSP (P = 0.0041) and BG_multi (P = 0.0050) groups, whereas no significant differences were observed for the BG_opacious (P > 0.05) or AS (P > 0.05) groups. Notably, the OC_multi (P = 0.0376) and OC_opacious (P = 0.0075) groups exhibited a significant increase in shear bond strength following thermal cycling.

The results of fracture surface analysis are shown in Table 1. Mixed and adhesive failure modes were observed; however, cohesive failures were not detected in any group. Overall, the proportion of adhesive failures was higher after 24 h of immersion, whereas more mixed failures were observed following thermal cycling.

**Table 1:** Distribution of failure modes after shear bond strength tests to dentin

Group	Storage^a^	Adhesive failure (%)	Cohesive failure (%)	Mixed failure (%)
BSP	24 h	20	0	80
TC	20	0	80
BG_multi	24 h	46.7	0	53.3
TC	20	0	80
BG_opacious	24 h	53.3	0	46.7
TC	13.3	0	86.7
OC_multi	24 h	66.7	0	33.3
TC	45.7	0	53.3
OC_opacious	24 h	53.3	0	46.7
TC	46.7	0	53.3
AS	24 h	46.7	0	53.3
TC	53.3	0	46.7
^a^24 h: Stored for 24 h in ultrapure water at 37°C; TC: Thermal cycling for 10,000 cycles between 5 and 55°C.

## DISCUSSION

This study tested the null hypotheses that incorporating organic composite filler, bioactive glass, or aluminosilicate glass into 4-META/MMA-TBB resin would not affect the curing time, flexural strength, or shear bond strength, and that bioactive glass would not affect the mineralization ability. Groups containing bioactive glass (BG_multi and BG_opacious) showed significant differences in curing time, flexural strength, and bond strength. However, little hydroxyapatite formed, indicating no clear improvement in mineralization ability. The addition of organic filler (OC_multi and OC_opacious) had significant effects on the curing time and bond strength, but not the flexural strength. Aluminosilicate glass (AS) significantly affected the curing time and flexural strength but had no effect on the bond strength. Thus, the null hypothesis was rejected for the following groups and properties: bioactive glass (curing time, flexural strength, and bond strength); organic filler (curing time and bond strength); and aluminosilicate glass (curing time and flexural strength). It was retained for bioactive glass (mineralization ability); organic filler (flexural strength); and aluminosilicate glass (bond strength). These results suggest that filler incorporation selectively affects certain physical and adhesive properties of 4-META/MMA-TBB resin.

A significant increase in curing time was observed in groups with bioactive glass, suggesting that the additives may influence the polymerization reaction. Gupta et al^10^ used DSC to investigate the curing behavior of GI cements containing various ceramic fillers, including silica, alumina, and zirconia. They reported that increasing the filler content prolonged the curing time. These findings are consistent with those of the present study. Additionally, Proença et al^20^ evaluated the physical properties of orthodontic bracket adhesives incorporating 5, 10, or 20 wt% of either 45S5 or NbG bioactive glass. They found that 45S5 increased the pH of the adhesive, whereas NbG induced neutralization. Because 4-META/MMA-TBB resin contains acidic monomers as adhesive components, an increase in pH may reduce the monomer reactivity, thereby inhibiting the TBB-initiated polymerization process. This suggests that bioactive glass may prolong the curing time by increasing the pH.

The three-point flexural strength decreased significantly after adding bioactive or aluminosilicate glass, suggesting that these additives may compromise the mechanical integrity of the resin. Yamamoto et al^25^ modified 4-META/MMA-TBB resin with 1.25, 2.5, or 5.0 wt% benzalkonium chloride (BAC) or cetylpyridinium chloride (CPC) and evaluated the flexural strength in accordance with ISO 4049. No significant differences were observed for groups with 1.25 or 2.5 wt% of either additive; however, the flexural strength of the 5.0 wt% BAC group was significantly lower than that of the control. These findings suggest that exceeding a certain concentration of additives may weaken 4-META/MMA-TBB resin. Additionally, Kim et al^13^ demonstrated that variations in the particle size and molecular weight of polymethyl methacrylate (PMMA) powder added to 4-META/MMA-TBB resin influenced the degree of polymerization and the bonding durability to titanium. Specifically, resins containing higher-molecular-weight PMMA exhibited reduced polymerization and lower bond strengths. These results suggest that the decline in mechanical properties observed in the present study may be attributed to the incorporation of bioactive or aluminosilicate glass.

The incorporation of functional materials into restorative systems has attracted increasing attention. Previous studies have demonstrated that organic composite fillers can enhance the mechanical properties and wear resistance of resin-based materials.^21,27^ In addition, bioactive glasses release Ca^2+^ and PO_4_^3−^ ions *in vivo*, which may promote dentin remineralization and inhibit caries progression.^7,26^ Meanwhile, aluminosilicate glasses can improve the flexural strength and surface hardness of GI cements and resin-based materials ^11,18^

The current study found no significant differences in mineralization ability when bioactive glass was added. Although GIXRD is capable of analyzing the outermost micrometers of a surface, no hydroxyapatite peaks were detected in any of the groups in this study. This indicates that the apatite phase, if present, was below the limit of detection. Kohda et al^14^ investigated the effects of bioactive glass on enamel remineralization. They added 0–50 wt% bioactive glass to 4-META/MMA-TBB resin, bonded the resin to enamel, and exposed the specimens to acidic conditions for 14 days. Higher concentrations of bioactive glass led to increased ion release (Ca, Na, Si, and B) and enhanced acid-neutralizing capacity. Notably, with 30–50 wt% bioactive glass, the enamel adjacent to the bonded area maintained significantly higher Knoop hardness than the control, suggesting that bioactive glass effectively inhibits demineralization. The relatively low concentration of BG used in this study (3 wt%), compared to the 30–50 wt% employed by Kohda et al, may explain the limited formation of hydroxyapatite crystals observed herein.

The shear bond strength to root surface dentin was evaluated after immersion in ultrapure water for 24 h at 37°C or after thermal cycling for 10,000 cycles between 5 and 55°C. Owing to limitations of the thermal cycler, the samples were stored or thermally cycled in ultrapure water; other media such as phosphate-buffered saline (PBS) or SBF could not be used. Nevertheless, ultrapure water is commonly used for similar tests.^25^ After thermal cycling, the shear bond strengths of the groups with organic composite filler were significantly higher than those of the groups with bioactive or aluminosilicate glass, suggesting that inorganic fillers like bioactive or aluminosilicate glass may negatively affect the bonding performance. Kohda et al^14^ evaluated 4-META/MMA-TBB resin containing 0–50 wt% bioactive glass and reported a trend of decreasing bond strength to enamel with increasing bioactive glass content. Similarly, Yamamoto et al^25^ assessed 4-META/MMA-TBB resin containing 1.25–5.0 wt% BAC or CPC; while no significant changes were observed in the shear bond strength to root dentin at concentrations of up to 2.5 wt%, after thermal cycling, the 5.0 wt% BAC group exhibited a significant reduction in bond strength compared to the control. In contrast, the OC_multi and OC_opacious groups in this study showed significantly higher shear bond strengths after thermal cycling compared with the 24 h immersion condition. This favorable performance can be explained by the presence of pre-polymerized organic filler in the 4-META/MMA-TBB resin. Previous studies have demonstrated that such organic fillers reduce polymerization shrinkage stress and improve the interfacial properties. In particular, Saeki et al^21^ reported that Bondfill SB, a commercial organic-filler-containing 4-META/MMA-TBB resin, had a significantly higher shear bond strength than a conventional 4-META/MMA-TBB adhesive. They ascribed this to a reduction in the interfacial inhibition layer and a more favorable stress distribution at the bonding interface.

These findings suggest that additives such as bioactive glass may reduce the bond strength of 4-META/MMA-TBB resin to root dentin. Fracture surface analysis revealed that the group stored in water for 24 h predominantly failed by adhesive failure, whereas mixed failures became more frequent after thermal cycling. This shift in failure mode is consistent with previous studies by Mitsui et al^17^ and Salagalla et al,^22^ who reported that increases in the adhesive interface strength over time or under thermomechanical loading can result in mixed failures extending into the substrate.

Several limitations of this study should be acknowledged. First, the experiments were conducted under *in vitro* conditions, which do not fully replicate the complexity of the *in vivo* environment. For example, in clinical practice, especially when treating older adults, ideal bonding conditions are often difficult to achieve due to factors such as bleeding and salivary contamination. In addition, bonding was performed on the root dentin of extracted human teeth after complete caries removal. Therefore, future studies should consider bonding to infected dentin to better reflect clinical scenarios. Furthermore, for older adults or medically compromised individuals with limited access to dental care, conventional restorative procedures may not be feasible.^6,9^ Thus, the experimental results of this study should be interpreted with caution, taking into account their divergence from real-world clinical conditions. Future studies should aim to identify the optimal bioactive glass concentration that promotes dentin remineralization without compromising curing time, bond strength, or mechanical properties. This could be accomplished by preparing specimens with incremental concentrations of bioactive glass and systematically evaluating their effects on each physical parameter. In addition, future investigations will assess the effects of immersion in PBS or SBF on shear bond strength to better reflect conditions that could influence bioactivity.

## CONCLUSION

In this study, the effects of various bioactive, inorganic, and organic fillers on the properties of 4-META/MMA-TBB resin were investigated. The incorporation of bioactive glass prolonged the curing time and reduced the flexural and shear bond strengths. Slight crystal formation was observed, but the extent of deposition was insufficient to clearly demonstrate hydroxyapatite formation or enhanced mineralization. In contrast, the incorporation of organic composite filler shortened the curing time and increased the shear bond strength without significantly affecting the flexural strength. The addition of aluminosilicate glass also reduced the curing time, but slight decreases in the flexural and shear bond strengths were observed. These findings suggest that organic composite fillers are the most suitable additives for 4-META/MMA-TBB resin-based restorative materials.

### Acknowledgments

The authors sincerely thank Sun Medical Co. for generously providing the materials used in this study and for their valuable technical support. This research was funded in part by Sun Medical Co., and further supported by a Grant-in-Aid for Research Activity Start-up from the Japan Society for the Promotion of Science (grant number 22K20986). The funding sources had no role in the design of the study; the acquisition, analysis, or interpretation of data; the writing of the manuscript; or the decision to submit it for publication. During manuscript preparation, the authors utilized ChatGPT (GPT-4o, OpenAI) to assist with English proofreading and to enhance readability. We would also like to thank Editage (www.editage.jp) for English language editing. All content was carefully reviewed and revised by the authors to ensure accuracy and clarity prior to submission. The authors take full responsibility for the final content of this manuscript.

### Clinical Relevance

Organic composite fillers reduce the curing time of 4-META/MMA-TBB resin while maintaining adequate bond strength, suggesting favorable clinical applicability for restoring root caries in older adults, particularly in situations where moisture control is challenging. In contrast, bioactive and aluminosilicate glasses reduce bond strength and mechanical properties, and should therefore be used with caution.

### Conflict of Interest

The authors declare that they have no conflict of interest.
